# Use of Nd:YAG laser in bruise resolution caused by an injectable dermal filler procedure

**DOI:** 10.1002/ccr3.5402

**Published:** 2022-03-16

**Authors:** Barry Dekeyser, Marijke Wellens, Iva Talaber

**Affiliations:** ^1^ Haar en Huid Kliniek Tessenderlo Belgium; ^2^ Fotona Ljubljana Slovenia

**Keywords:** bruising, dermal fillers, ecchymosis, injection, laser therapy, Nd:YAG laser

## Abstract

Injectable dermal fillers are in high demand. Patients undergoing this treatment are frequently burdened by bruising in the days following the procedure. Bruises associated with dermal fillers usually resolve spontaneously within 10 to 14 days, but patients want shorter downtime. We present a case of a bruise treatment with Nd:YAG laser consisting of three sessions in two day intervals, where the bruise resolved after seven days from the start of laser treatment. To date, the reports on laser therapy for bruise resolution are mostly limited to intense pulsed light and pulsed dye laser, and this case report contributes to the demonstration of Nd:YAG laser efficacy for this indication.

## Graphical abstract

Bruising or ecchymosis is a frequent adverse effect of injection and a primary concern to many patients considering injectable dermal fillers. Nd:YAG laser treatment can accelerate bruise resolution time and can therefore be used by medical practitioners to decrease downtime in patients undergoing this popular aesthetic procedure.

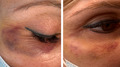

## 1 INTRODUCTION

The downtime following a cosmetic procedure is an important consideration for patients.^1^ Bruising is a common adverse event encountered with injectable dermal fillers.^1^, ^2^ with reported incidence of 24^3^ or even 68^4^ percent. Nevertheless, demand for injectable dermal fillers is high, with many procedures performed yearly. Although bruising is mostly an aesthetic concern, patients can be strongly impacted as it may limit the patient's social activities and the possibility to discreetly undergo this type of cosmetic procedures. A bruise, also known as an ecchymosis, is a reddish or bluish discoloration of the skin arising from extravasation of blood from ruptured blood vessels.^5^ Use of the right instruments for injectable dermal fillers can reduce bruising. Blunt‐tipped microcannulas are recommended by some practitioners as larger cannulas and needles are more likely to transect blood vessels and cause bruising.^6^ Patients with thin and fragile skin and older patients with slower repair mechanisms are more prone to bruising and slower recovery.^7^

Bruising will normally resolve spontaneously within 10–14 days; however, it can persist up to three weeks after the aesthetic procedure.^7^ Methods for treating post‐procedure ecchymosis are limited. Application of cold compression^7^ following injection is used to reduce the risk for bruising via vasoconstriction.^6^ Various nonprescription topical and oral agents are also used. There are reports in the literature that pre‐ and/or post‐procedure application of *Arnica montana*, vitamin K8, or bromelain leads to less bruising following cosmetic surgery^2^, ^8^ or increase in the speed of resolution^2^, ^9^ although these do not demonstrate consistent results.^10^, ^11^

As bruising is caused by extravasation of blood and subsequent release of hemoglobin to the interstitial space, selective photothermolysis with light/laser therapy can be used to treat bruises,^12^ with hemoglobin serving as the chromophore.^13^ There are sufficient number of studies suggesting that pulsed dye laser (PDL)^10^, ^12^, ^14^, ^15^ or intense pulsed light (IPL)^5^ accelerates bruise resolution. The Nd:YAG wavelength is highly absorbed by the hemoglobin in the tissue. Therefore Nd:YAG laser can also be used to treat bruises;^16^, ^17^ however, there is limited information on the use of this wavelength in the literature.

## 2 CASE DESCRIPTION

A patient with Fitzpatrick III skin type came to the office on September 1 (2020) to receive an injectable filler treatment, which consists of hyaluronic acid filler placement in the deep dermal plane at the zygomatic region. The patient reported a history of bruising after dermal fillers. In December 2019, the patient underwent treatment with calcium hydroxyapatite in the perioral zone which resulted in excessive bruising.

The practitioner decided to use a cannula technique in favor of a sharp needle since this is the preferred technique in the case of patients prone to bruise formation or prolonged bleeding. The cannula technique is safe and tolerable. To make an entry point for the 25 gauge cannula, a relatively large 23 gauge puncture needle is used. By puncturing the skin, a blood vessel was hit by the needle. Subcutaneous bleeding occurred immediately that resulted in a bruise. The procedure was stopped and a firm pressure was applied for several minutes. Vitamin K cream (Auriderm XO) was applied for 24 h to reduce the bruise formation. Ice was recommended for 24 h.

At follow‐up consultation after three days, the bruise showed no signs of resolution. To aid in bruise resolution, laser therapy was employed on the 4th day after the initial dermal filler procedure. Patient was treated with 2–3 passes of 1064 nm Nd:YAG laser with the R33 handpiece (Fotona, Slovenia) and the following settings: 4mm spot size, 20 J/cm^2^ fluence, 0.6 msec pulse duration, and 2 Hz frequency. Cooling (Zimmer Cryo 5, Zimmer MedizinSystems, USA) was applied during the procedure. Treatment was repeated three times in two day intervals. The patient was monitored for adverse effects and pain associated with laser therapy.

## 3 RESULTS

**FIGURE 1:**
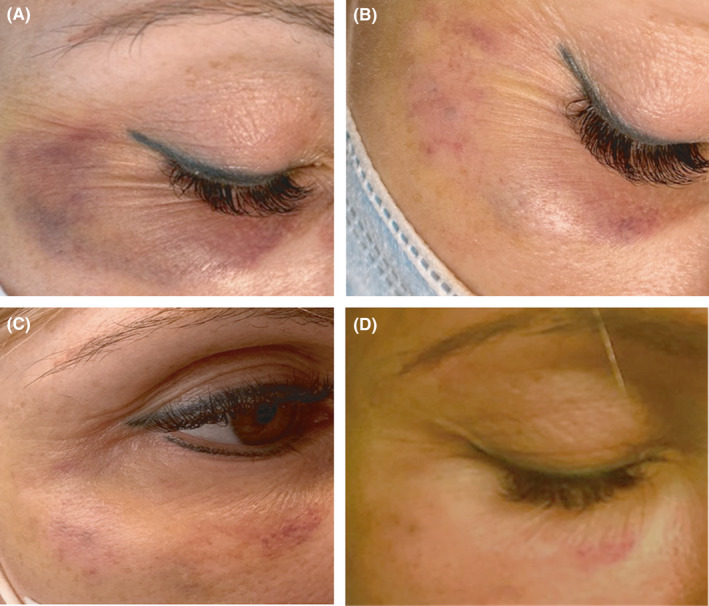
Gradual resolution of a bruise during seven days of Nd:YAG laser treatment. The bruise occurred after a dermal filler procedure. (A) Before first laser session. (B) Two days after first laser session. (C) Two days after second laser session. (D) Two days after third laser session. Courtesy of Marijke Wellens, RN

The bruise resolved gradually in the course of treatment, as seen in the photographs documenting the case (Figure 1A–D).

## 4 DISCUSSION

Lasers have been used to treat post‐injectable bruising for a number of years, but many medical professionals are still not aware of this possibility.^18^ The most frequently used laser modality for ecchymosis resolution is the PDL laser. One of the first studies^12^ on post‐surgery ecchymosis performed with PDL reported that the best response was achieved when laser treatment was administered three days after operative procedure or later. At this time, the extravasated red blood cells may be more susceptible to laser destruction due to migration from deeper to superficial layers of dermis, and their subsequent lysis and release of hemoglobin into the interstitial space. In addition, at this time, there is less tissue edema and inflammation, so more of the laser energy can reach the target chromophore.^12^ In accordance with this, a later study^15^ found no significant effect of PDL treatment on bruise resolution when administered shortly (30 min) after bruise induction. It has also been reported^14^ that the best effects can be achieved on bruises with a pronounced erythematous and/or violaceous component, suggesting that PDL intervention is most effective if initiated when hemoglobin predominates. Accordingly, less improvement has been observed^10^ in patients with deeper or older ecchymosis that showed an initial paler green, yellow, or brown coloration. Moreover, the efficiency of laser treatment seems to depend on the stage of bruise evolution and the cause of the bruise. Bruises after nonsurgical cosmetic procedures, for example dermal fillers, are typically more superficial and associated with less tissue inflammation and edema compared to post‐surgery bruises.^14^

In our case, Nd:YAG laser treatment with 0.6 msec pulses was administered to a patient with a Fitzpatrick III skin type presenting a superficial reddish bruise with limited edema (Figure 1A). Therapy with low fluence was employed four days after the initial dermal filler procedure. The bruise resolved in seven days from the onset of laser treatment (Figure 1B–D). No adverse effects were noted, and the treatment was not painful for the patient. Although the information about Nd laser treatment of bruises is limited, one study^18^ has reported significant results in bruise clearance in patients of all Fitzpatrick skin types using similar pulse duration (0.65 msec). They treated darker areas of the bruise with low fluence and lighter areas of the bruise with higher fluence. In another study,^16^ Nd laser was specifically chosen over the PDL to treat purpura and ecchymosis in patients with darker skin types (Fitzpatrick IV‐VI) due to comparatively deeper penetration, less absorption by epidermal melanin, and limited thermal injury to the epidermis and upper papillary dermis of the Nd:YAG wavelength. Compared to our case, this study used considerably higher fluence and longer pulse duration and reported notable bruise resolution after 24 h.^16^

Depending on the severity and the stage of the bruise, the parameters for the Nd:YAG laser treatment may be adjusted and a higher fluence considered. However, caution is needed in treatment of ecchymosis with any laser source because the accumulation of erythrocytes represents a very high density of chromophores that may lead to bulk heating.^19^

## 5 CONCLUSIONS

Low intensity short pulse Nd:YAG 1064 nm laser showed efficacy in reducing the bruise resolution time without any adverse effects. More cases would be needed to establish the optimal protocol of this promising therapy.

## CONFLICT OF INTEREST

Iva Talaber is an employee of Fotona d.o.o., the manufacturer of a medical device used in the study. Other authors have no conflicts of interest to declare.

## AUTHOR CONTRIBUTIONS

Author 1: did conceptualization of study, interpretation of clinical data, and writing of the manuscript. Author 2: did conceptualization of study and interpretation of clinical data. Author 3: did interpretation of clinical data, writing of the manuscript, and figure preparation.

## ETHICS APPROVAL

All procedures performed in studies involving human participants were in accordance with the ethical standards of the 1964 Helsinki Declaration and its later amendments or comparable ethical standards.

## CONSENT

The patient signed an informed consent form after understanding the nature of the trial.

## Data Availability

The data that support the findings of this study are available from the corresponding author upon reasonable request.
